# Understanding the Role of Clinical Champions and Their Impact on Clinician Behavior Change: The Need for Causal Pathway Mechanisms

**DOI:** 10.3389/frhs.2022.896885

**Published:** 2022-07-13

**Authors:** Alexandra L. Morena, Larissa M. Gaias, Celine Larkin

**Affiliations:** ^1^Department of Psychology, University of Massachusetts, Lowell, MA, United States; ^2^University of Massachusetts Chan Medical School, Worcester, MA, United States

**Keywords:** champion, implementation, strategies, barriers, behavior change

## Abstract

**Background:**

The clinical champion approach is a highly utilized implementation strategy used to mitigate barriers and improve outcomes of implementation efforts. Clinical champions are particularly effective at addressing provider-level barriers and promoting provider-behavior change. Yet, the specific causal pathways that explain how clinical champions impact provider behavior change have not been well-explicated. The current paper applies behavior change models to develop potential causal pathway mechanisms.

**Methods:**

The proposed mechanisms are informed by previous literature involving clinical champions and empirically supported behavior change models. These models are applied to link specific attributes to different stages of behavior change and barriers for providers.

**Results:**

Two unique pathway mechanisms were developed, one that explicates how providers develop intention to use EBPs, while the other explicates how providers transition to EBP use and sustainment. Clinical champions may promote *intention development* through behavioral modeling and peer buy-in. In contrast, champions promote *behavioral enactment* through skill building and peer mentorship.

**Conclusion:**

Clinical champions likely play a critical role in reducing provider implementation barriers for providers across various phases of behavior change. The proposed pathways provide potential explanations for how clinical champions promote provider behavior change. Future research should prioritize empirically testing causal pathway mechanisms.

## Introduction

Evidence-based practices (EBPs) can help reduce suffering and prevent premature death in those experiencing mental health conditions (1, 2). Yet, EBPs are underutilized among mental health clinicians and organizations (3, 4). Many individuals seeking mental health care do not receive empirically supported treatments (4) and there is a substantial gap between establishing empirical support and integrating an EBP into routine care (5, 6). Implementation science research aims to bridge this gap by investigating factors that promote and impede uptake of best practices while also developing and testing strategies to promote successful implementation (4). Implementation strategies are techniques or methods used to enhance the uptake, implementation, and sustainment of a clinical program or practice (7). While the field has focused on developing and testing strategies, mechanisms explaining how strategies operate need to be more thoroughly explored (8). Causal mechanisms explain the processes through which implementation strategies generate a desired effect or outcome and will allow researchers to better select implementation strategies based on their project goals (9, 10).

In the current paper, we examine causal pathways through which one such implementation strategy, the identification and preparation of clinical champions address and reduce provider-level barriers to uptake of EBPs. The “identifying and preparing clinical champions” strategy was selected due to its described impact on provider behavior change across implementation efforts (11). While this strategy is regularly utilized in various implementation efforts, less is known about the underlying mechanisms that may be contributing to its success. To inform the development of the proposed causal pathway mechanisms, the current paper provides a theoretically informed review of the current clinical champion literature to outline relevant attributes and describe common responsibilities and processes champions engage in.

## What (AND WHO) are Clinical Champions?

Clinical champions are individuals who are dedicated to supporting, advocating for, and spearheading an implementation initiative, and who overcome resistance that may occur at the organizational level (12). They have an intrinsic interest to implement change and use their position to motivate others (13–15). Previous research has referenced clinical champions of a specific topic (e.g., hand-washing champions), discipline (e.g., nurse champion), or broadly within an organization or implementation effort (e.g., executive champion) (15).

Research involving clinical champions have begun to identify attributes that may impact an implementation effort (14, 16, 17). Clinical champions have been described as having strong communication and mentorship skills. Strong communication and mentorship skills involves processes including collaborating with others, advocating for change, ability to negotiate as well as educate and facilitate learning (15, 17). Strong communication and mentorship skills can facilitate buy-in by conveying their conviction and positive perceptions about the initiative to their peers (14, 17, 18). Champions can also effectively tailor messages to different audiences to maximize engagement and buy-in (15, 16, 18).

Previous research has also emphasized clinical champion's EBP knowledge and competency (14, 17, 19). Clinical champions often emerge due to their knowledge and previous experience which, in part, is how clinical champions promote EBP adoption within their clinical environment (13, 17). Additionally, as clinical champions serve as a resource for providers to develop EBP competency, effective champions must be knowledgeable, experienced, and have strong self-efficacy to effectively educate others (15, 19). Through their knowledge and expertise, clinical champions may also engage in skill-sharing, and promote benefits of integrating the EBP into clinical practice (13, 14, 17).

Clinical champions have also been described as being deeply embedded in their clinical setting. Embeddedness in the clinical setting means that a clinical champion has frequent face-to-face to contact with their peers as well as leadership and are regularly present on the frontlines (i.e., the point of change) (16, 20). This embeddedness results in a robust understanding of their setting's culture and workflow (16, 20). This embeddedness may allow the clinical champion to model integration of an EBP into their daily workflow as well as providing education and support to peers (16, 17). A clinical champion's presence in the clinical setting can have substantial impact on implementation; results from Rycroft-Malone et al. (18) found clinical champions' embeddedness allowed for a more grass-roots approach to implementation, which yielded better EBP uptake. This embeddedness also relates to clinical champions' institutional savvy (16), which allows them to effectively navigate the complex social hierarchies and culture that exists within their setting/organization. This allows clinical champions to identify points of potential resistance and leverage their relationships/influence to overcome resistance (16). Not only are clinical champions dedicated to their clinical practice (i.e., frequently on the frontlines), but they are also dedicated to their role and readily embrace change (15, 17, 19). This dedication to both the innovation and overall implementation effort may provide clinical champions with the drive needed to overcome resistance in their clinical setting, which may be key to their impact on both the implementation effort and provider behavior change (16, 17, 19).

Arguably the most impactful attribute of clinical champions is their standing as informal leaders in their clinical organizations (16, 19, 20). Informal leaders are regarded as highly influential individuals who do not hold positional authority but are highly respected due to their expertise, trust, and relationship-building capabilities (21–23). The power of informal leaders is defined as “one's ability to initiate action and ensure the desired outcomes are produced” (21). Informal leaders take time to emerge, as a provider needs to develop both the technical expertise and trust from others before being deemed influential (22). Clinical champions may be perceived as implementation experts by their peers and, due to being highly respected, are then able to actively engage them in implementation efforts (16, 19, 20). Although clinical champions may hold formal leadership positions (17), effective clinical champions are typically informal leaders (16, 17). The power being referenced throughout this paper is in reference to this “subtle” power informal leaders possess. Clinical champions' influence, power, and relationship-building capabilities have been considered crucial to implementation success (14, 16, 19). Additionally, clinical champions have been described as highly respected and valued individuals within their clinical organization who routinely establish meaningful relationships with their peers (14, 16, 17). It is through these meaningful relationships that clinical champions can effectively engage their peers in implementation efforts. Thus, the current paper defines informal leadership/influence as the following: an individual who is highly respected by their peers who establish strong and meaningful relationships with them and are viewed as a credible and reliable source of information and skill-sharing (14, 16, 19, 24, 25). It is important to note that clinical champions may overlap substantially with another group of influential individuals within healthcare organizations, local opinion leaders. Local opinion leaders are individuals who are respected informational sources who have influence over others' decisions and behaviors (24, 25). Both clinical champions and local opinion leaders are viewed a fellow peers who have an in-depth understanding to a provider's day-to-day experience while also being viewed as credible and reliable source of information (24, 25). Clinical champions also overlap with early adopters, individuals that readily adopt new ideas, are typically solution-oriented, and embrace innovation (26, 27). Yet, there are also areas where clinical champions may diverge from these groups. Clinical champions are unique to local opinion leaders, as champions act through charismatic leadership, as opposed to organizational norms and structure (24). Instead, clinical champions may take an active role toward shifting organizational climate to be more amenable to change (16). Additionally, clinical champions are unique to early adopters, as early adopters do not necessarily take the responsibility for promoting and spearheading change initiatives in their settings (28).

## Clinician-Level Implementation Barriers

Clinical champions may be particularly relevant to an implementation effort due to their potential to influence the behavior of other frontline providers within their clinical setting. Frontline providers are essential to an EBP implementation effort because they have autonomy to make clinical decisions and can also utilize their power and influence to persuade others to adopt new practices (29). Each provider has their own unique set of values, interests, and ways of enacting the organizational culture (29), which may facilitate or impede their use of EBPs. The Consolidated Framework for Implementation Research [CFIR, (29)] model has identified provider-level determinants that impact an implementation initiative, including knowledge and attitudes about the implementation initiative (i.e., a specific intervention), self-efficacy (e.g., belief in one's own ability to carry out implementation goals), individual stage of change (e.g., progressing toward becoming a skilled and enthusiastic implementer), and identification with their organization.

Provider-level barriers have been identified across multiple studies and are generally defined as attitudinal or behavioral and involve cognitive or psychological processes that impede or prevent a target behavior from occurring (4). Commonly cited examples include knowledge, attitudes, and self-efficacy (3, 10). Barriers surrounding EBP knowledge, competency, or utility in a particular treatment context can impact providers' intentions to enact an EBP and/or their ability to deploy or sustain the EBP (4). Such barriers can persist and limit a provider's ability to deploy or sustain an EBP, even when they intend to do so (4, 30).

In addition to provider-level barriers, the CFIR model describes implementation determinants at other levels including the inner and outer setting (29). The outer setting refers to how external policy, economic, or social context impact implementation (29). In contrast, the inner setting refers to the setting-specific culture, norms, and general characteristics (i.e., geographic location, size) (29). Together, barriers that exist at different levels of the implementation setting may interact and in turn impact the implementation effort's outcome and success (29, 31). For example, results from Mosson et al. (32) found that managers in smaller organizations or those in rural areas had difficulty with implementation due to barriers that impacted them at the individual level, such as lack of training or preparedness. This occurred, in part, due to the organization's geographical location, causing training opportunities to be infrequent (32).

While it is important to acknowledge barriers at other levels (e.g. organizational, cultural), provider-level barriers may be important to target due to their amenability to change (33). Targeting an individual's beliefs, attitudes, or behaviors may be more feasible than addressing barriers at the system level. Previous research suggests that when EBP attitudes and beliefs are targeted *via* social persuasion tactics, EBP utilization and fidelity increased (33). Previous research has also emphasized a key factor to successful EBP implementation is most successful when line-level clinicians and organizational members are willing and ready to change (34). Thus, focusing on individual-level attitudes, beliefs, behaviors, or practices may allow for improved design, implementation, and generalizability of strategies to explicitly target such barriers (35, 36).

## Implementation Strategies and the Need For Mechanism Research

To reduce implementation barriers and promote uptake of EBPs in healthcare and other settings, implementation scientists have developed and tested implementation strategies (5, 10). Implementation strategies can target different populations including patients, clinical providers, stakeholders, and policymakers, as well as different phases (e.g., preparation, delivery, sustainment) and levels (e.g., individual, inner setting, outer setting) of the implementation effort (10). Given the range of implementation strategies that were being used and reported in the literature, Powell and colleagues (12) compiled a taxonomy of 73 common and discrete implementation strategies, the Expert Recommendations for Implementing Change (ERIC). The ERIC taxonomy was created using an expert panel and aimed to unify and standardize how implementation strategies are referenced and defined in the literature. Although the ERIC taxonomy has effectively allowed for standardized and well-defined implementation strategies, our understanding of how such strategies operate in real-world implementation efforts is limited (9, 10). Per the ERIC taxonomy (12), the implementation strategy “identify and prepare champions” is defined as, “preparing individuals who are dedicated to supporting, marketing, and driving through an implementation, overcoming indifferences or resistance that the intervention may provoke within an organization.” This strategy is a multi-faceted approach that prioritizes fostering stakeholder relationships, providing mentorship to line-level providers, and overseeing the implementation process (i.e., creating implementation plans, addressing barriers, engaging stakeholders) (17, 37, 38).

An area of the implementation strategy literature that needs to be more thoroughly developed is causal pathway mechanisms (9, 10). Causal pathway mechanisms outline the processes through which the implementation strategy of interest operate to impact one or more implementation outcomes (8). By better understanding how strategies operate to yield the desired outcome, researchers will be able to better select and modify implementation strategies to enhance both clinical and implementation outcomes (9, 10). Understanding processes of implementation strategies will help explain why a certain strategy is effective or ineffective and in what contexts they operate best (8). Most importantly, understanding processes of implementation strategies will allow for better strategy selection which in turn will better address barriers in the specific implementation context (8). Per Lewis et al. (8), if there is no intentionality behind strategy selection, a suboptimal or less potent strategy may be selected and applied to implementation efforts.

Existing frameworks, such as the Behavior Change Wheel (39), have been developed to examine how behavior change is most likely to occur given intervention components/functions and under certain policy conditions. However, linkages between certain implementation strategies and specific behavioral changes have not been as well explicated. Better understanding mechanisms will require implementation scientists to move beyond simply describing whether a strategy was effective toward developing testable theories that explain relationships between implementation strategies and outcomes as well as allow for outcome prediction (9, 10).

The current paper proposes two causal pathway models for one specific implementation strategy: identifying and preparing clinical champions (5). This implementation strategy was intentionally selected due to its potential for addressing provider-level barriers. When implementation researchers and practitioners were asked to rank the implementation strategies that would best address each of the CFIR barrier domains, identifying and preparing clinical champions was the most consistently rated strategy across all provider-level barriers (11). This implementation strategy leverages the influence and respect clinical champions hold within their clinical setting and the interpersonal relationships they form with other providers, which are particularly salient in the context of provider behavior change (40). By building and engaging in interpersonal relationships within their organizational setting, clinical champions can effectively mitigate provider-level barriers that impact a provider's intention and motivation as well as their ability to deploy an EBP.

## Clinical Champions and Provider Behavior Change

While previous literature emphasizes clinical champions' power and influence as a crucial factor in this strategy's effectiveness (14, 16, 17), exactly how clinical champions change provider behavior has yet to be empirically examined. A conceptual model outlined by Shea (19) posits that a clinical champion's commitment to an implementation effort, as well as their experience, self-efficacy, and performance, promote peer engagement with a clinical champion, which directly influences a clinical champion's impact. A key component of this model is the need for clinical champions to facilitate buy-in from their peers (19). Clinical champions can promote buy-in by being knowledgeable, trustworthy, and reputable within their organization (16, 19). Clinical champions can also influence their peers through various forms of power, including expert power (i.e., ability to influence behavior due to skills, knowledge, and abilities), referent power (i.e., impact individual behavior through being well-liked and admired), and informational power (i.e., promote behavior change *via* exchange of knowledge) (41, 42). Expert and referent power are considered power that leads to social dependent change, as they are cultivated *via* how other individuals view the person. Expert power develops through being perceived as credible and trustworthy, while referent power is cultivated through being admired and well-liked (42). Informational power is considered a power that leads to socially independent change, as disseminating information between the power source and target requires acceptance of knowledge, which the target continues to absorb and apply on their own following knowledge exchange (43).

To outline how peer engagement with a clinical champion can reduce provider-level barriers to implementing EBPs and facilitate behavior-change, we apply two social-cognitive models, the Theory of Planned Behavior [TPB; (44)] and the Health Action Process Approach [HAPA; (45)]. The Theory of Planned Behavior [TPB; (44)] describes how an individual develops intentions to perform a specific behavior and posits that their motivation or intention to engage in a behavior impacts the likelihood of them doing so (44). Intention development is influenced by an individual's attitudes toward the behavior (i.e., individual's evaluation of the behavior), subjective norms (i.e., perceived social expectations or pressures to engage in a specific behavior), and their perceived behavioral control (i.e., perceived ability to adequately perform the behavior) (40, 46). Extending beyond intent and motivation, the HAPA model is a social-cognitive dual-phase model that describes the processes needed to transition from intent to action (47). The HAPA model contains two phases to explain executing a particular behavior: a motivational (intention formation) and volitional phase (planning for behavior enactment and action) (45, 48). A key component of the HAPA model is the distinction between individuals who are engaging in pre-motivational processes that lead to developing intent and those who are engaging in post-intentional volitional processes or behavioral enactment (45). During the motivational phase, an individual is considered a “pre-intender” as they are developing intent to adopt a new behavior (45, 49), similar to the TPB. Once intent is established, an individual enters the volitional phase, which is broken into two separate classifications, “intenders” (i.e., individuals intending to perform the behavior in question) and “actors” (i.e., individuals already engaging in desired behavior) (45). These unique stages of behavior change align well with the types of individual-level barriers providers face, which also can relate to developing intention to use EBPs (i.e., attitudes and knowledge) and deploying EBPs (i.e., planning) (4, 30).

These models were selected to inform the proposed causal pathways due to their relevance to understanding adult behavior change and their routine application in implementation science to address the research-to-practice gap (50–52). These theories clearly explicate individual-level factors that impact behavior change and can also account for the role of others within the implementation context, such as clinical champions, in influencing both implementation intentions and action (e.g., through social norms). These models also have been supported empirically (53–55). The TPB has been routinely used in previous implementation science research aiming to investigate clinical provider's intentions to adopt EBPs (2, 40). Additionally, the HAPA model provides a unique contribution beyond the TBP by also accounting for behavioral engagement and other factors that address the intention-behavior gap (56). Whereas other adult-focused behavioral change theories describe constructs that predict overall behavior intention, the HAPA model can also outline potential causal pathway mechanisms relevant to providers who actively transition from intention to behavioral enactment and then need to sustain that behavior enactment over time (51, 56).

Establishing hypothesized causal pathway mechanisms will advance our current understanding of how this specific implementation strategy, identifying and preparing clinical champions, influences provider delivery of EBPs. Understanding these mechanisms is crucial to advancing the scientific literature and will allow for better understanding of existing implementation strategies, strategy development, and appropriate strategy selection for use in specific implementation contexts (9, 10). The proposed causal pathways explain how clinical champions impact providers in various stages of the behavior change process: pre-intenders (i.e., those developing intent to perform the desired behavior), intenders (i.e., individuals intending to perform the behavior), and actors (i.e., individuals who have engaged in the desired behavior) (45, 49).

### Pathway 1: Pre-intenders

**Figure 1:**
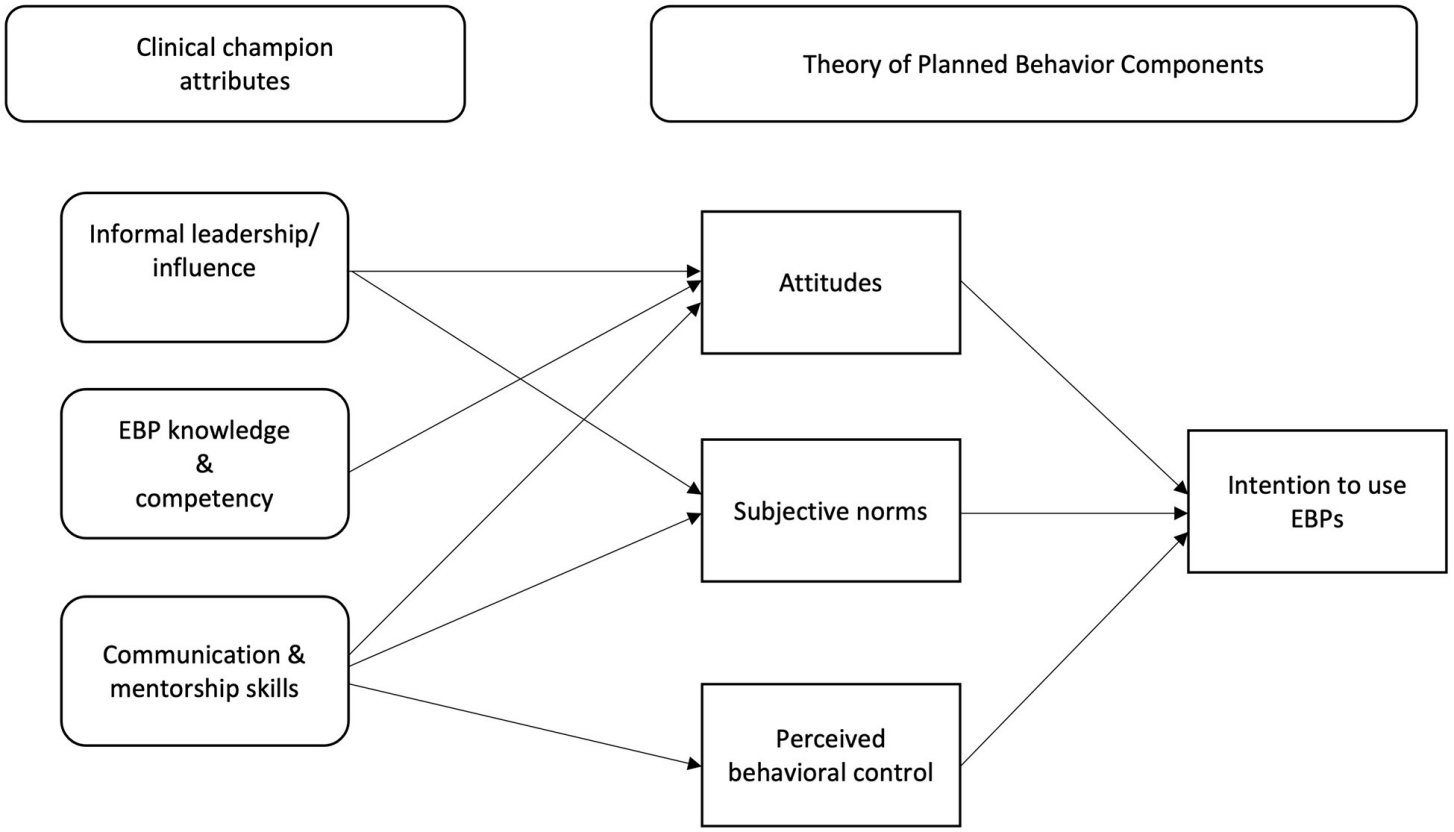
Causal pathway for development of intention to use EBPs.

The provider-level barriers most relevant to pre-intenders include attitudes and beliefs about an EBP, perceived utility of the EBP in clinical practice, and lacking confidence in EBP utilization (4, 29, 30). Strong intent must be formed prior to behavioral enactment and is considered a necessary precursor to behavioral action, with intent being regarded as a bridge between key motivational processes, specifically setting a specific goal (i.e., identifying which novel behavior to adopt) and pursuing said goal (i.e., behavioral enactment) (45). Strong behavioral intentions are formed when an individual's attitudes toward said behavior are positive, they perceive that their peers endorse said behavior (i.e., establishing subjective norms), and that they can adequately execute the behavior (40). Please see Figure 1 for visual representation of the proposed causal pathway discussed in detail below.

Subjective norms are crucial to target, as subjective norms have been identified as the strongest predictor of intent to utilize EBPs (40). Subjective norms are not changed through one provider alone, they must become the collective values, beliefs, and behaviors of the clinical setting. Through cultivating multiple one-on-one communication channels advocating for EBP utilization, it is possible that clinical champions promote a shift in norms at the setting-level, which can also impact behavioral or attitudinal shifts at the individual-level. Clinical champions may promote intention development for pre-intenders through shifting a provider's **subjective norms** due to their *informal leadership/influence* (i.e., being respected, viewed as credible/reliable source of information, build strong relationships) (2, 24, 25, 40) and *strong communication and mentorship skills* (i.e., collaborating, advocating, and facilitating learning) (15, 17).

Through their informal leadership/influence, clinical champions are perceived as a credible source and engage in social comparison. Being perceived as a credible source, providers are likely to absorb and apply information about the EBP as said information is coming from a trusted “expert” source (16, 17). As a credible source, clinical champions also engage in social reinforcement, a crucial process that routinely occurs in the clinical setting, as providers want to determine whether their clinical practices are like others (24, 57). Providers report higher intent to utilize an EBP when a respected provider within their organization approves of them using or reinforcing the behavior (2, 40). Other findings suggest that intent to engage in a specific behavior is higher when messages approving the behavior came from a respected colleague as opposed to supervisors (2). Such findings suggest that in this context, messaging from influential informal leaders impacts behavior more profoundly than when such messages come from formal leadership figures, who may not have direct experience delivering the practice. Through informal leadership/influence, champions exert referent (i.e., being well-liked and admired) and expert power (i.e., ability to influence behavior due to skills, knowledge, and abilities (41, 42), due to being highly respected and reliable sources of information.

Clinical champions may also promote shifts in subjective norms through their *strong communication and mentorship skills*. Strong communication and mentorship skills involves negotiating and collaborating with others as well as advocating for change and the ability to educate and present information to others (15, 17). As previously mentioned, communicating about the EBP (e.g., its benefits to patient care) can help facilitate peer buy-in to utilize the EBP (14, 17, 18). Clinical champions promote buy-in not only by providing accurate information about the EBP, but by also tailoring their message to specific provider groups (15, 16, 18). Tailoring messaging may allow for the clinical champion to enhance peer buy-in because they are able to address concerns about utilizing the EBP and address other barriers (e.g., knowledge) in a way that can resonate strongly with multiple and varied providers. Thus, the clinical champion may aid in establishing an overall consensus regarding the EBP's importance which ultimately can shift behavior of a specific individual (15, 16). Through these strong communication and mentorship skills, it is possible that clinical champions exert expert power (i.e., ability to influence behavior due to skills, knowledge, or abilities) and informational power (i.e., promote behavior change *via* knowledge exchange) (41, 42) as they utilize their communication skills to promote knowledge and skill-building in their peers (14, 16).

Clinical champions also promote intention development through changing pre-intenders' attitudes toward the EBP itself. In addition to changing subjective norms, a clinical champion's *informal leadership/influence* can also facilitate change in provider's attitudes. Again, by being a credible source, highly respected, and having strong interpersonal relationships with peers, when clinical champions advocate for and promote use of an EBP in clinical practice, their opinions regarding how to deliver high-quality care are likely to be taken seriously by their peers (14, 16). Additionally, clinical champions *EBP knowledge and competency* may also be highly influential on provider attitudes toward the EBP. Clinical champions must have high levels of knowledge and competency about the EBP they're trying to implement to effectively prepare their fellow providers to integrate the EBP into their own practice (14, 17). Through disseminating this knowledge, clinical champions may be able to effectively address misconceptions about the EBP, address questions providers may have about the EBP, all of which may facilitate development of positive outcome expectancies, an essential component of the motivational phase of the HAPA model (45). Clinical champions with high levels of EBP knowledge and competency are likely to regularly promote and demonstrate the EBP's utility in the clinical context, which may also promote changes in provider's attitudes toward the EBP (14, 16, 17, 58). A clinical champion's knowledge and competency allows them to exert both expert and informational power (i.e., promote behavior change *via* knowledge change) as they utilize their own knowledge to persuade peers to adopt the EBP (41, 42).

Clinical champion's may also impact provider's attitudes toward the EBP through their *strong communication and mentorship skills*. By being exposed to accurate information from their peers, it is plausible that providers can develop more positive attitudes about the EBP itself, especially when said information is coming from a well-liked and respected source. Per the Diffusion of Innovation theory [DOI, (59)], an individual's decision to adopt a specific practice is, in part, influenced by acquiring knowledge about the innovation and being persuaded that the practice will benefit them (59, 60). Clinical champion's strong communication and mentorship may allow for an individual obtaining the necessary knowledge about the EBP to deem it beneficial while also being persuaded to adopt the innovation from a highly respected peer within their social network. Through these processes, clinical champions are again exerting their expert and informational power.

Lastly, clinical champions may also enhance pre-intenders' perceived behavioral control through their *strong communication and mentorship skills*. Strong communication skills utilized by clinical champions may enhance pre-intender's perceived behavior control, as a common practice of clinical champions includes individualized training and providing tailored feedback (14–16, 58). Such behaviors require a clinical champion to be able to effectively collaborate with peers, advocate for EBP use, and provide individualized feedback. Similarly, strong mentorship skills have been operationalized as willingness to facilitate learning, collaboration, and to provide education (61). Strong mentorship skills may allow for clinical champions to enhance pre-intender's perceived behavioral control because they received the necessary education and support needed to develop more confidence in their ability to apply the skills needed to utilize the EBP effectively. It is important to note a clinical champion's mentorship may be most impactful when they are committed to the implementation effort and training (15, 19).

### Pathway 2: Intenders and Actors

In contrast to pre-intenders, intenders have developed the necessary intent needed to enact the behavior but have not transitioned from intent to action (i.e., enacting the desired behavior) (45, 49). Provider-level barriers commonly experienced by intenders are those related to EBP deployment (i.e., lack of planning and skills) (4). Intenders have successfully transitioned into the volitional phase (i.e., the phase in which someone develops plans to act or has transitioned to action) but have yet to become an actor. The HAPA model suggests that action and coping planning are critical for translating intention into action for intenders (45). Previous research suggests that action planning aids individuals to effectively identify cues for behavioral engagement as well as develop actionable steps needed to effectively execute the behavior of interest (47, 62). In contrast, coping planning entails identifying potential barriers that may impact behavior execution and developing plans to mitigate them (47).

#### Intenders

For intenders, clinical champions' promote behavior change through aiding in action and coping planning, due to their *embeddedness in the clinical setting* (i.e., being present on the front lines, frequent face-to-face contact with peers) (16, 45). Frequent presence on the frontlines allows clinical champions to regularly and readily engage with clinical providers to support their integration of EBP into their routine practice, which in turn promotes skill building and planning (16, 17). On a similar note, frequent face-to-face contact with fellow providers also facilitates skill building and planning, as doing so allows for providers to easily access support and mentorship from clinical champions when needed (16, 17). Face-to-face engagement when providing education and skill-building is known to be more impactful than passive education strategies, like treatment guides or websites (12, 17)]. Through their embeddedness, clinical champions may also be exerting their referent [i.e., being well-liked and admired (41)] and expert power, as they leverage their relationships and ability to cultivate strong social bonds to facilitate EBP adoption.

Clinical champion's *informal leadership/influence* also promotes action and coping planning. To develop skills and outline actionable steps to integrate EBPs into clinical practice, providers need an individual whom they respect and view as a credible source of information whom they can turn to for advice, education, and support (14, 16, 19, 23). As clinical champions wield influence and are viewed as informal leaders, it is possible that intenders trust and rely on the clinical champion as a resource to aid in planning and skill development (16, 17, 58). Additionally, any mentorship, training, and education provided by the clinical champion is taken seriously by their peers due to their informal leadership/influence (14, 16).

Lastly, development and enhancement of provider's action and coping planning skills can be impacted by clinical champion's *strong communication and mentorship skills* (i.e., negotiating, collaborating, advocating, and educating/facilitating learning). Through their strong communication skills, clinical champions may increase clinicians' self-efficacy and over time can lead to sustained used of the EBP during clinical care (17, 63). Strong communication and mentorship skills also aids providers in engaging in action planning. Action planning involves breaking down the EBP or intervention into easy to execute steps, meaning it is crucial for a clinical champion to be an effective communicator (56), so these steps are easily understood by the intender. Previous research in both medical and education contexts have investigated the benefits of peer-to-peer coaching and skill sharing, with findings suggesting that such teaching approaches promote the use of evidence-based practices, reflection on current workplace practices, and collaborative discussion (64, 65). Additionally, medical settings that applied peer coaching practices have found peer coaching improves educators' skill transferability, confidence, and overall satisfaction (64).

A clinical champion's *EBP knowledge and competency* may also promote intender's development of action planning, coping planning, and achieving behavioral enactment (19). Skills in these domains relate to both engaging in the behavior of interest as well as performing the clinical champion role itself (19). Thus, clinical champions will be most impactful on intender behavior change when they themselves are knowledgeable about the EBP as well as have high self-efficacy and confidence in their ability to use the EBP as well as lead and mentor other providers (19, 66). Through their previous experience and high self-efficacy, clinical champions themselves have already engaged in all action planning components, including defining EBP steps, outlining how to execute each step of the EBP during clinical care, and identifying potential barriers (56). It is possible that clinical champions themselves have also engaged in coping planning by identifying barriers that occurred in their own clinical practice and developed steps to mitigate them (56).

#### Actors

Lastly, clinical champions can also aid a third group of providers, actors (i.e., individuals who have begun actively enacting the desired behavior), in maintaining their behavioral enactment and continuing to use the EBP. Although actors have successfully enacted the desired behavior (i.e., EBP use), they still experience barriers and can benefit from support. A common barrier cited by providers is that implementing EBPs consistently and with fidelity is challenging (4). Relevant volitional phase processes for actors include maintenance self-efficacy (i.e., perceived ability to maintain desired behavior) and action control (i.e., self-monitoring, awareness of standards, effort) (45, 49). Through clinical champions *strong communication and mentorship skills*, actors can receive continuous mentorship and training/education regarding EBP utilization, which in turn allows for development of maintenance self-efficacy and consistent EBP use (56, 63).

**Figure 2:**
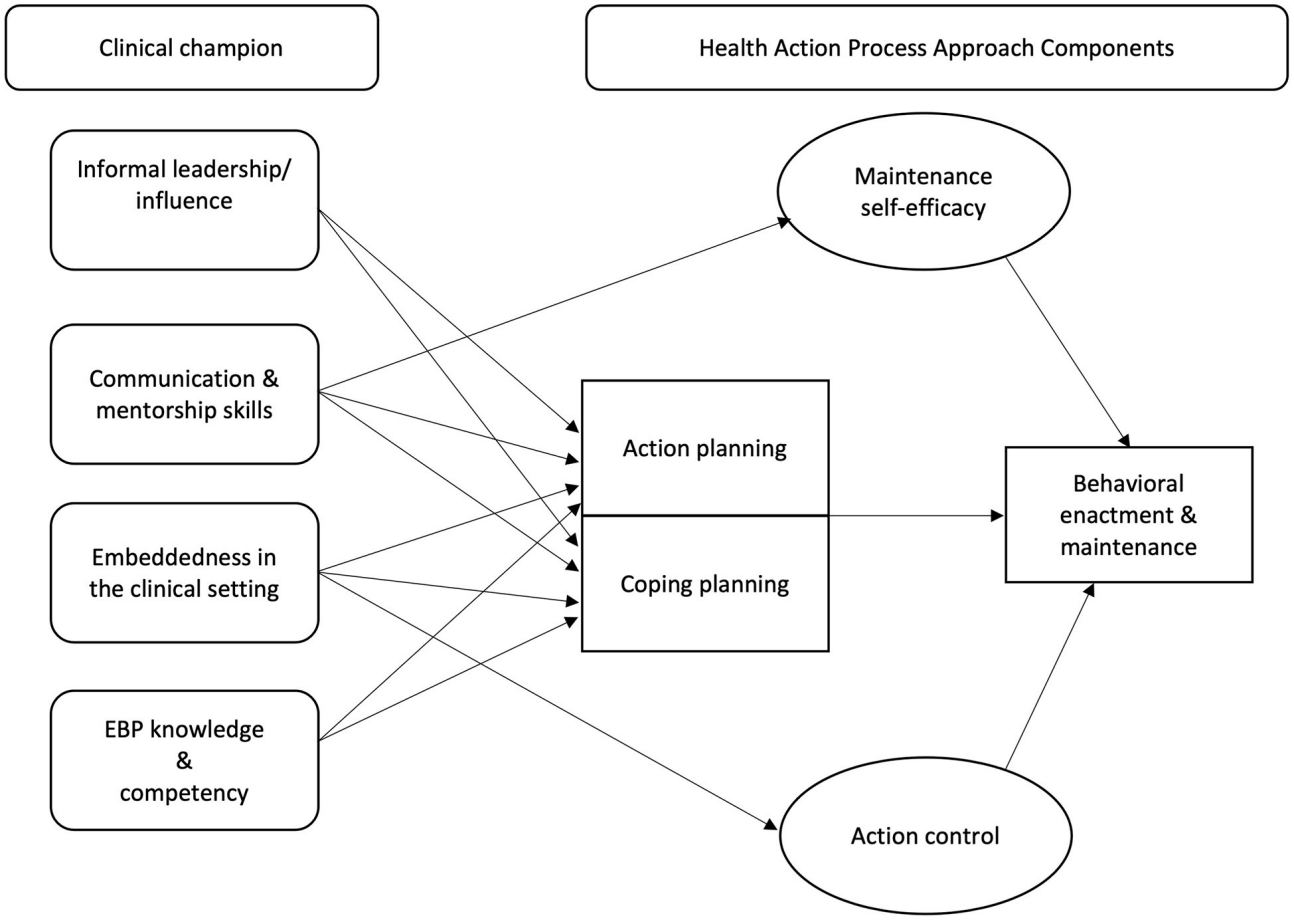
Causal pathway for development of behavioral enactment and maintenance.

Clinical champions' can also impact actor's action control through their *embeddedness in the clinical setting* (i.e., presence on the frontlines and frequent face-to-face contact with peers). By being embedded in the clinical setting, clinical champions hold actors accountable to consistently utilize the EBP when relevant. Previous research also suggests that due to their embeddedness, clinical champions often engage regularly in compliance monitoring (16, 58). Holding actors accountable can promote development of action control, as actors will begin to self-monitor and be more self-aware of EBP use in their clinical practice. See Figure 2 for visual representation of this causal pathway.

### Pathway Summary

In summary, clinical champions largely operate through social influence. Social influence involves one individual to evaluate a respected figure's own perceptions of the specific innovation (59, 67). Social influence is interwoven with diffusion, a social process in which members of a specific social system communicate an adoption decision for a particular practice (59, 67). Clinical champions and similar roles may impact diffusion of an innovation through their adoption and advocating of the EBP, as doing so may influence the social system to shift their normative practices (i.e., accepting the innovation or practice) (67). Such roles may also aid in accelerating one's decision to adopt the EBP (60, 67). Adoption decisions are often accelerated when an influential person within a social system adopts the innovation themselves and communications that decision across the social network (67).

Secondly, clinical champions wield social influence due to their resemblance to their peers because they too are often fellow line-level providers. Potential adopters of a specific innovation often seek judgment from a trusted and respected “expert” peer (67), and this interpersonal communication is known to be more impactful when there are professional similarities between communicators (60). Per the Diffusion of Innovation theory [DOI, (59)] there are five factors involved in an individual's process in deciding to adopt an innovation: acquiring knowledge about the innovation, being persuaded the innovation is beneficial, engaging in activities that may impact a choice, incorporating the innovation into daily workflow, and seeking reinforcement about their decision (59, 60). Clinical champions may be tapping into all these factors, as they often provide education to peers about the EBP, attempt to persuade individuals to adopt the EBP, validate one's utilization of the EBP and decision to adopt, and provide the support needed for the individual to incorporate the EBP into their routine patient care (2, 14, 16).

### How Causal Pathways Inform Clinical Champion Selection

These causal pathways outline how a clinical champion, once identified, can influence provider behavior change. However, these pathways do not include the selection or identification of clinical champions themselves. Despite research that has described common characteristics of clinical champions, less is known regarding how clinical champions are selected, identified, and trained (17, 68). A review conducted by Wood et al. (17) suggests that clinical champions are typically selected/recruited or emergent/self-designated. Selected champions are providers who typically had prior experience utilize the EBP or intervention, whereas emergent champions take on this role due to an inherent interest, expertise, or conviction for the EBP or intervention (17). In contrast, a review of clinical champion utilization in nursing homes by Woo et al. (68) found that clinical champions were either selected by clinical leadership or selection was not adequately described. Similar trends have also been observed regarding clinical champion training, with many studies not adequately describing how clinical champions are trained or stating whether they were trained at all (17, 68). The few studies that have described clinical champion training report common training modalities including in-person workshops about the intervention, online training modules, and education about implementation and leadership strategies (37, 38, 69).

The pathways outlined above can provide suggestions for the recruitment, identification, and training of clinical champions. When attempting to identify potential clinical champions, it is important to first observe the clinical setting and take note of the interpersonal relationships that exist (21). Seek the provider who is not only able to form strong interpersonal connections with their peers, but also the one to whom others gravitate toward for guidance and support, as these providers have influence over others and may be strong informal leaders (21, 23). Previous research suggests that for behavioral modeling and knowledge transfer to be most impactful, the individual must identify and relate to the individual modeling the desired behavior (70, 71). If the clinical champion selected is not representative of most providers being targeted, this implementation strategy may not generate the desired effect (i.e., provider behavior change). Thus, when selecting clinical champions, it is important to seek providers who display these informal leadership attributes, such as expertise, trust, and influence (16, 21, 23). Although not much research has been focused on the selection of clinical champions, research examining the selection of key opinion leaders can provide some insight into best practices. For example, (72) suggest that local opinion leaders can be identified *via* self-selection (i.e., individuals volunteer due to personal reasons or strong desire to serve), staff selection (i.e., project staff select leaders based on observations), “judge ratings”, (i.e., fellow line-level providers select local opinion leaders rather than formal leadership staff). This final identification method may be well-suited for selecting clinical champions, as it relies on fellow providers and is easy to implement in larger-sized settings (72).

### Additional Considerations for Future Research

In the current paper, we have hypothesized causal pathways rationalized with well-established scientific behavior change models to explore how and why clinical champions facilitate provider behavior change. These pathways explain how clinical champions can mitigate provider-level barriers commonly experienced by providers who are in various stages of the behavior change process (i.e., pre-intenders, intenders, and actors). Explicating potential causal pathways is one step toward better understanding implementation strategies and enhancing their impact as they connect implementation strategies to behavior change theories and go beyond describing general effectiveness (9, 10). While these causal pathways outline how clinical champions prompt provider behavior change, the next step is to empirically test them; this can inform our understanding of which implementation strategies operate best in certain contexts (9, 10). A review by Lewis et al. (8) found that mediation models were the most common approach for examining how a specific implementation strategy impacted the relationships between implementation outcomes, with implementation determinants acting as a mediator. A mediation model design could examine whether the presence of a clinical champion and their embodiment of the characteristics described above impacts the outlined mechanisms (e.g., subjective norms, action planning) and whether those mechanisms in turn impact EBP use and fidelity. Moderated-mediation models could also examine how clinical champions could interact with features of the implementation context at the organizational or community level, as discussed further below. Future research should test the proposed causal pathway models in different contexts and settings to observe how these proposed mechanisms operate across different healthcare settings.

#### Contextual Considerations

It is important to discuss the importance of contextual factors, specifically the inner (i.e., organization's size, location, culture, climate) and outer (i.e., political, economic, social factors) setting, as clinical champions are impacted greatly by the environments in which they are engaging and trying to transform (29, 32). As implementation efforts occur in complex systems that have various levels of influence ranging from the system- to patient-level, it is important to understand how these factors interact with implementation strategies and impact implementation (73). Barriers at both the inner and outer setting levels can impede leadership's impact on achieving successful implementation efforts (32, 73). Understanding how organizational context interacts with implementation strategies is needed to further the field's understanding of how implementation strategies operate and yield desired outcomes. It is possible that implementation strategies can optimize uptake and application of EBPs by either making the inner and outer setting more amenable to implementation or by adapting the EBP to better fit within the organization (73).

Common outer setting barriers include organization and policy makers interest and willingness to support the implementation initiative and other policy-related barriers (e.g., financial disincentives) (16, 32). Implementation efforts occurring within organizations who supported the implementation initiative *via* funding or trainings (i.e., providing trainings for how to utilize the EBP) typically had more impactful implementation outcomes and success (32). Supportive environments allow for implementation leaders to provide staff with ample high-quality training opportunities and the financial support needed to invest in the implementation process (32). Results from (16) observed that clinical champions working in an environment with substantial outer context barriers, such as financial disincentives, resulted in unsuccessful implementation and even de-implementation due to the inability to overcome such barriers. In environments where outer context barriers are prevalent and/or outside the sphere of a clinical champion's reach, this implementation strategy may not be the most impactful (16).

Inner setting factors are described as structural (e.g., size, geographic location) and modifiable (e.g., culture, climate, readiness for implementation, communication) (29, 32). Inner setting barriers that are relevant to clinical champion's impact include implementation climate (32, 73, 74), staff resistance to change (16), and relationships between clinical champions and organization leadership (16, 32). As clinical champion's leverage their influence and ability to form strong interpersonal relationships (16, 17), for settings with suboptimal or low-quality relationships between line-level staff and formal leadership or administrators, clinical champions may experience substantial difficulties achieving success due to an inability to establish buy-in or overcome resistance from leadership (16, 32). Strong positive relationships between clinical champions and implementation leadership are crucial, as leadership is critical to a successful implementation effort (75). As leadership provides support, feedback, and guidance to implementation (75), clinical champions with poor relationships with leaders may not be able to overcome certain barriers.

Implementation climate [i.e., extent to which an organization's policy, practice, and culture is amenable to implementing innovative and new practices (66)] is highly relevant and impactful regarding clinical champions and their impact on both the implementation effort and provider behavior change. Implementation climate has been identified as a key indicator of both staffs' prolonged use of a specific innovation as well as the quality in which the innovation is delivered (74). A supportive implementation climate in which the organization is open to new ideas (66) and both leadership and line-level staff prioritize using EBPs creates optimal conditions for clinical champions (and other implementation leaders) to successfully facilitate implementation (32, 66). When implementation climate is poor, clinical champions may experience challenges related to communicating and educating line-level providers about the EBP, which will hinder their overall impact and implementation success (16).

#### Equity

Future research should also prioritize understanding clinical champions in the context of health equity and disparity research. To move toward equitable health outcomes, implementation science needs to proactively tailor and modify implementation strategies and EBPs to address health disparities (76, 77). This includes how implementation strategies can facilitate equitable implementation across healthcare services (76, 77). To our knowledge, previous research has yet to explicitly investigate how clinical champions impact health disparity implementation efforts and should be explored in future work. For example, it will be important to identify whether certain characteristics of clinical champions are particularly critical (and whether there might be additional necessary characteristics) for implementing practices that are explicitly aimed at reducing biases and inequities within the healthcare system.

In addition, related to the discussion above regarding context, it is important to consider the heterogeneity of resources that exist across the healthcare system and how such differences impact clinical champions. Populations experiencing health inequities often receive services in low-resource settings, which can be impacted by staff shortages, higher staff turnover, and lack of funding (77, 78). Thus, these settings require additional implementation resources to be successful, including both financial resources and leadership supports (77, 79). Low-resource settings often experience barriers at the inner setting level (i.e., staff turnover, resource shortages, competing demands), which may reduce the impact clinical champions have. When testing implementation strategy mechanisms, an explicit focus should be given to the likelihood for the strategy to have its intended impact across varied settings, without implicitly assuming that equitable outcomes will be facilitated through the use of the implementation strategy (80).

It is also critical to consider the potential for inequities to arise throughout the process of recruiting, selecting, and training clinical champions. Enacting implementation strategies without a focus on equity could allow disparities to emerge in both implementation and clinical outcomes (80). Although it is important for clinical champions to be well-networked and hold influence within their organizations, power within organizations can replicate hierarchies or systems of power and privilege reflected in society more broadly (63, 81). Perceptions of core clinical champion characteristics, such as trust/respect, communication, and leadership are influenced by socio-cultural positionality, including, but not limited to, race/racism, gender/sexism, and ability/ableism (82). Biases can emerge when identifying certain providers as leaders for example [e.g., (83, 84)] and therefore the role of culture and positionality should not be ignored when identifying clinical champions. Power imbalance and hierarchies present in both healthcare teams and organizations can have negative impacts on implementation. Power imbalances that plague healthcare organizations include those related to communication (i.e., receptivity and responsiveness from leadership), trust and respect, as well as role allocation (i.e., lack of recognition or delineation of duties) (81). For clinical champions, imbalances related to communication may impede collaboration and educating peers about the EBP, which may reduce their overall effectiveness. Power imbalances in the context of trust and respect may be the most detrimental to clinical champions, as without trust and respect clinical champions yield no influence as informal leadership (16, 21, 34). Additionally, if there is a lack of trust and respect between a clinical champion and formal leadership, clinical champions may experience irreconcilable barriers to implementing change.

### Limitations of Clinical Champions

It is also important to address potential weaknesses of the clinical champion implementation strategy. While the current paper focuses on clinical champion attributes and the mechanisms through which these attributes exert their effects, not all clinical champions display the described characteristics, embrace their role, or achieve success. A common challenge experienced by clinical champions involved limited bandwidth and competing demands that prevent them from fulfilling their clinical champion role duties (14, 16, 58). Clinical champions are often also providing patient care, which is their primary responsibility as a healthcare provider. Such competing demands could impact a clinical champion's ability to provide adequate training/education about the EBP and monitor usage and fidelity (14, 58). Another weakness of clinical champions noted in previous literature is lack of ownership of the initiative or role (14, 16). Not all clinical champions prioritize the EBP being implemented in their organization or may have other responsibilities they are more dedicated to (14, 16). As embracing the initiative and using their drive and commitment to motivate others to adopt the EBP, clinical champions lacking this dedication may not be as successful. Clinical champions may experience social barriers or weaknesses that impede them from fulfilling their duties, such as lacking influence (16) and navigating boundaries with their fellow peers (58). Part of the clinical champion's role is to observe their peers and provide them with feedback or point out ways in which they can improve their utilization of an EBP, some clinical champion's may feel conflicted or uncomfortable navigating boundaries with their peers or hierarchies that exist within their clinical setting (58). Lastly, it is important to acknowledge that clinical champions alone may not be sufficient in achieving system-level change (15, 16). Clinical champions may be particularly effective in environments with engaged and supportive leadership, training supports, and a positive implementation climate.

It is also possible that clinical champions may lack certain attributes that can be cultivated with further training and support. For example, a clinical champion may be identified who, although is a strong informal leader who wields influence, may not have EBP-specific expertise. In such cases, EBP-specific trainings should be provided to allow for expertise to be established (38). In contrast, some clinical champions may not possess strong informal leadership skills, in which case leadership trainings should be provided (37). As the field continues to develop an understanding of this implementation strategy, further research is needed to explore how we can better support and prepare clinical champions so they can be most effective.

## Conclusions

Clinical champions likely play a critical role in reducing provider implementation barriers for clinicians across various phases of behavior change (e.g., pre-intenders, intenders, and actors). Clinical champions promote the development of intention by displaying significant knowledge about the EBP, behavioral modeling of the EBP's utility, and establishing peer buy-in. In contrast, clinical champions aid intenders to transition into behavioral enactment through skill building which can promote sustained usage of the EBP. This paper contributes to the gap in scholarship outlining potential mechanisms of implementation strategies and can lead to future research testing such mechanisms.

## Author Contributions

AM devised the main conceptual ideas and outline, developed the pathway mechanisms, guided and supervised by LG, and drafted mechanisms figures with guidance from CL. AM and LG wrote the manuscript, with consultation and input from CL. All authors contributed to the article and approved the submitted version.

## Conflict of Interest

The authors declare that the research was conducted in the absence of any commercial or financial relationships that could be construed as a potential conflict ofinterest.

## Publisher's Note

All claims expressed in this article are solely those of the authors and do not necessarily represent those of their affiliated organizations, or those of the publisher, the editors and the reviewers. Any product that may be evaluated in this article, or claim that may be made by its manufacturer, is not guaranteed or endorsed by the publisher.
